# Comparative Study of Percussion Drilling in Glasses with a Femtosecond Laser in Single Pulse, MHz-Burst, and GHz-Burst Regimes and Optimization of the Hole Aspect Ratio

**DOI:** 10.3390/mi14091754

**Published:** 2023-09-07

**Authors:** Pierre Balage, Manon Lafargue, Théo Guilberteau, Guillaume Bonamis, Clemens Hönninger, John Lopez, Inka Manek-Hönninger

**Affiliations:** 1 Université de Bordeaux-CNRS-CEA, CELIA UMR 5107, 33405 Talence, France; 2 AMPLITUDE, Cité de la Photonique, 33600 Pessac, France

**Keywords:** ultrafast laser processing, femtosecond GHz-bursts, laser–material interaction, glasses, percussion drilling

## Abstract

In this contribution, we present a comparative study on top-down drilling in sodalime glass, with a femtosecond laser operating in single-pulse, MHz-burst and GHz-burst modes, respectively. We investigate the hole depth, drilling rate, and hole morphology for these three regimes while keeping the same experimental conditions. We demonstrate that, for both burst regimes, the burst length has to be adapted for optimizing the hole depth. In the GHz-burst regime, the lower the ablation rate the longer the holes. The three drilling regimes lead to different hole morphologies, where the GHz-burst mode results in the best hole quality featuring glossy inner walls and an almost cylindrical morphology. Furthermore, we obtain crack-free holes, the deepest measuring 3.7 mm in length and 25 µm in entrance diameter corresponding to an aspect ratio of 150, which is the highest aspect ratio reported thus far with femtosecond GHz-burst drilling to the best of our knowledge.

## 1. Introduction

In the last two decades, ultrafast laser technology has widely enriched the industry owing to its versatility and its unique capabilities for the micro-processing of transparent dielectric materials [1]. Many applications have emerged in the literature, such as index change [2], 3D data storage [3,4,5,6], waveguides and diffraction gratings writing [7,8,9,10], and bottom-up drilling [11,12], as well as zero-kerf and dust-free glass cutting [11].

Thanks to the ultra-short interaction time, femtosecond lasers allow for a highly localized and intense energy deposition [13,14]. However, the throughput may not meet industrial requirements due to the small amount of material being processed during the interaction with each single pulse. An interesting strategy for increasing the productivity of femtosecond laser processing is spatial beam shaping. For instance, Bessel beam shaping has been studied for glass cutting [15,16,17], as well as nano-hole drilling [18], in-volume nanoscale modifications [19], and high-aspect-ratio drilling [20]. This technical solution allows for an elongated, very localized, and homogenous energy deposition. Gaussian beams are also used for high-aspect-ratio drilling with elongated spatial beam shaping, for instance by using a spatial light modulator [21].

An alternative approach to spatial beam shaping is the use of temporal beam shaping by spreading the pulse energy into a burst containing several femtosecond pulses instead of applying one single highly energetic pulse. The so-called burst regime has been mainly investigated for metals’ and semiconductors’ processing [22,23,24,25]. The take-home message of these four studies is that the use of bursts can, with the appropriate set of parameters, enhance the energy deposition and thus increase the removal rate. These studies also suggest that the use of MHz bursts of femtosecond pulses may not be optimal as the pulse-to-pulse delay within a burst is still longer than the heat-relaxation time of the materials. To overcome this limitation, the GHz-burst mode has emerged with a pulse-to-pulse delay of the same order of magnitude as the heat-relaxation time in dielectrics [26]. Some recent studies dealing with GHz-burst processing on dielectrics for milling applications have highlighted the same trend as the one observed on metals and silicon [27,28,29]. These first observations were recently confirmed by a comparative study dedicated to surface ablation on fused silica in MHz- and GHz-burst regimes [30]. It appears that the removal rate increases with the number of pulses per burst but at the expense of the surface quality. These studies assume that the GHz-burst interaction relies on thermal accumulation during the burst owing to the high repetition rate of the pulses within the burst. Other works have reported on combining MHz- and GHz-burst strategies in a biburst mode for ablation [31,32]. Very recently, we investigated the percussion drilling process in glasses [33,34] and in sapphire [34] in GHz-burst mode applying pump-probe shadowgraphy and thermal imaging [34]. Furthermore, we have shown that efficient drilling was even possible at fluences where the individual pulses within the burst have a fluence clearly lower than the ablation threshold of the material, which underlines that this processing mechanism profits from beneficial thermal accumulation. A very recent study has reported on the effect of the energy repartition within the GHz-burst on the drilling process [35].

In this study, we report on a comparison of top-down percussion drilling in sodalime glass for the repetitive single-pulse regime, as well as the MHz-burst and GHz-burst regimes, respectively, using the same laser source. We investigated the evolution of the hole depth as a function of the number of repetitive single pulses or bursts, as well as the hole morphology and the inner-surface quality via measuring microscopy. Furthermore, we optimized the parameters for reaching extreme hole geometries with an aspect ratio of up to 150 in fused silica in the GHz-burst mode by tuning the burst repetition rate and the numerical aperture.

## 2. Materials and Methods

The experiments were carried out using a modified industrial laser system based on a Tangor 100 from Amplitude, which is described in Reference [34] and produces pulses of 530 fs pulse duration at a wavelength of 1030 nm. This flexible laser system allows for a precise optimization of the drilling parameters such as the intraburst repetition rate, burst repetition rate, burst energy, number of pulses per burst, or burst shape. In this study, we compared different strategies of temporal beam shaping and focused on the influence of the intraburst repetition rate, as well as the number of pulses per bursts. The experiments were carried out with the laser system operating with repetitive single pulses, with MHz bursts at a 40 MHz intraburst repetition rate and a number of pulses per burst ranging from 2 to 8, and with GHz bursts at a 1.28 GHz intraburst repetition rate with a number of pulses ranging from 50 to 400. Using the same laser system and drilling setup for all experiments allowed us to lead a fairly robust comparative study.

**Figure 1:**
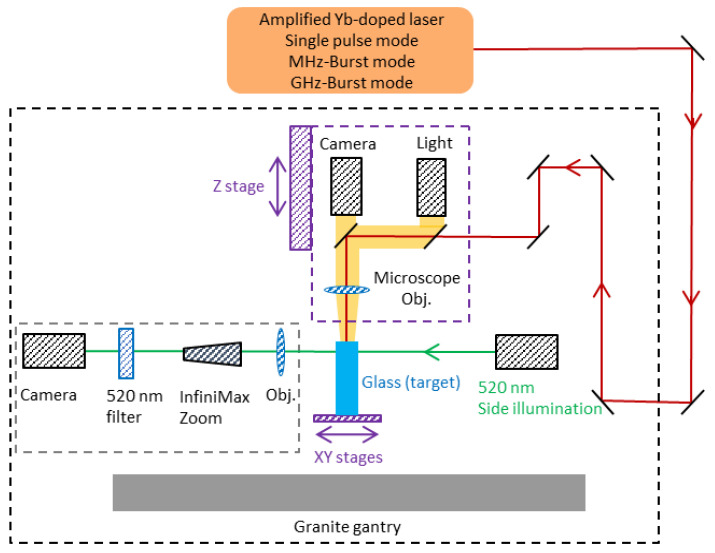
Experimental setup for the drilling experiments with sideview in situ observation.

The holes were drilled by focusing a Gaussian beam on the surface of the glass samples thanks to a microscope objective Mitutoyo Plan NIR Apo 5X with an effective numerical aperture of 0.14, resulting in a measured spot size of 9.3 µm at 1/e^2^. The spot size was measured using a 10X-homemade calibrated system with an uncertainty of ±0.66 µm. Thanks to a topview Basler CMOS camera, a white light source, and two dichroic mirrors, we can visualize through the focusing objective and accurately set the position of the laser focus at the front surface of the glass samples as schematically depicted in Figure 1. During the drilling, we use a sideview system composed of a green diode emitting at 520 nm for illumination and a Basler camera (Basler acA1920-25mu) coupled with a long-distance microscope (InfiniMax KX with MX-6 objective) for real-time imaging. The latter is equipped with a 520 nm-bandpass filter in order to directly visualize through the samples and not being blinded by the processing laser wavelength. The focusing head is mounted on a Z-motorized stage (VP25X, MKS Instruments), whereas the sample is fixed on a motorized XY-monolithic stage (One-XY60, MKS Instruments). The XYZ-stages and the laser gate are controlled by the DMCpro software (Direct Machining Control, Vilnius, Lithuania). The latter includes an autofocus function allowing for a precision in the focus position down to a few microns, reducing the positioning uncertainty and leading to the same conditions for all drilling experiments. The workstation has a granite base and gantry ensuring a high stability and an excellent repeatability of the experiments.

An optical measuring microscope (MF-B1010D, Mitutoyo) is used for ex situ imaging and accurate measurements of the hole depths and diameters with a precision of ±2.2 µm + 0.02·L, with L being the measured length in mm. We used a 20X objective for hole depth measurements and a 50X objective for fine observations of the inner wall morphologies. The study on the different operating regimes was carried out using sodalime glass, whereas we used fused silica for the optimization of the drilling parameters for obtaining extreme hole aspect ratios.

## 3. Results and Discussion

### 3.1. Drilling of Sodalime Glass: Comparison between Repetitive Single Pulses, MHz-Burst, and GHz-Burst Regimes

In this section, we present a comparative study of different temporal beam shaping strategies. The standard regime of repetitive single femtosecond pulses is applied and compared to the MHz-burst and the GHz-burst regime containing different numbers of pulses per burst (ppb). We investigated the evolution of the hole depth and the hole morphology in sodalime while increasing the number of bursts or pulses for the single-pulse regime (SPR). The number of bursts or pulses (in SPR) was varied from 1 to 10,000 for all operating regimes. Both the single pulse and the burst repetition rates were set to 1 kHz. Indeed, from a prior study [34] we know that higher repetition rates produce an extended and undesirable heat-affected zone surrounding the hole. Finally, even a collapse of the glass under its own weight arises, closing the hole if one increases the repetition rate too far. Obviously, the repetition rate limits depend on the material, and in sodalime the appearance of the heat-affected zone is around 20 kHz burst repetition rate in the GHz-burst mode. Therefore, the choice of a pulse or burst repetition rate of 1 kHz, respectively, ensures that drilling experiments are well below this limit in GHz-burst mode. The pulse (burst) energy in the single-pulse mode and MHz-burst mode can be adjusted from 50 µJ to 140 µJ, and in the GHz-burst mode in the range from 89 µJ to 286 µJ. As has been shown, high energy values are preferable as they lead to deeper holes [33]. Therefore, we chose the maximum energy that is possible in all three operational regimes, which is 140 μJ, in order to realize the whole study with the same total energy exposure and to be able to highlight the differences between the three regimes. As a consequence, the single-pulse mode corresponds to repetitive pulses of 140 µJ energy, whereas the MHz- and GHz-burst modes correspond to bursts containing N pulses carrying an energy of 140/N µJ each.

**Figure 2:**
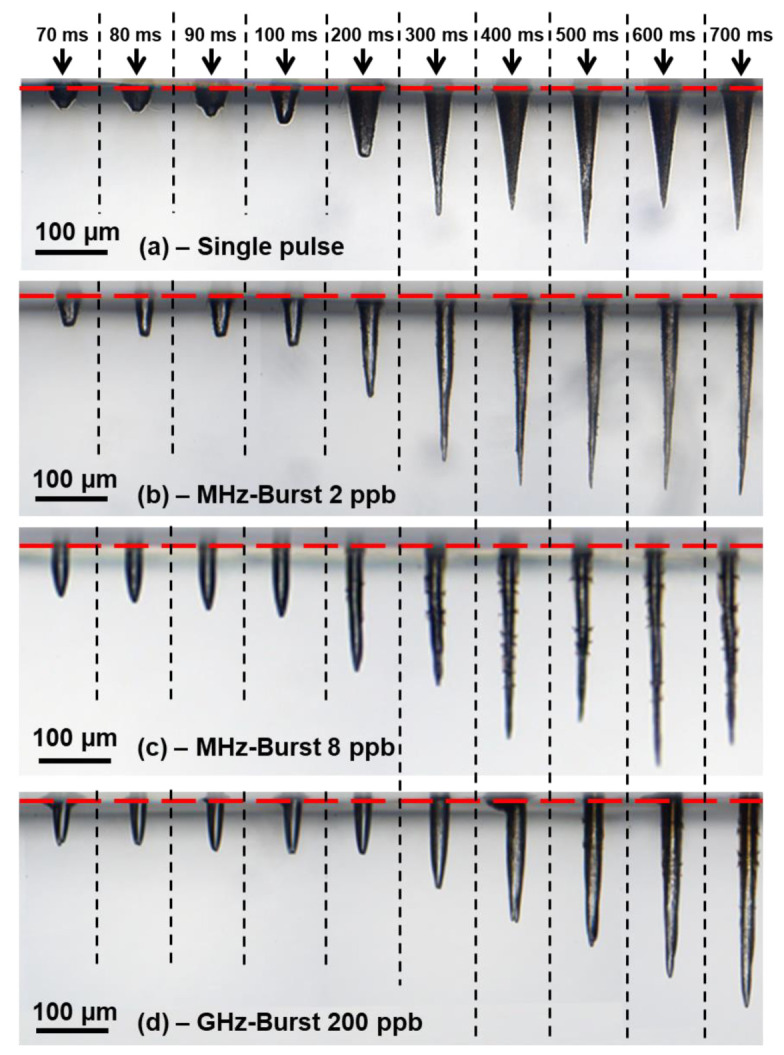
Sideview microscope images of the holes drilled in sodalime with a drilling time ranging from 70 ms to 700 ms with repetitive single pulses of 140 µJ (**a**), with 140 µJ-MHz bursts of 2 ppb (**b**) and 8 ppb (**c**), and with 140 µJ-GHz bursts of 200 ppb (**d**). The red dashed line indicates the sample surface. The laser comes from the top.

Figure 2 displays microscope images of the holes obtained for the single-pulse regime, upper image (a); for the MHz-burst regime with 2 and 8 ppb, middle images (b) and (c); and for the GHz-burst regime, lower image (d) with 200 ppb. These images were obtained for short drilling times ranging from 70 ms to 700 ms, corresponding to 70 bursts (pulses) to 700 bursts (pulses). The resulting hole depth ranges from 34 µm to 197 µm in the single-pulse regime, from 49 µm to 274 µm in the MHz-burst regime (2 ppb), from 80 µm to 308 µm for MHz bursts (8 ppb), and finally from 32 µm to 280 µm for the GHz-bursts with 200 ppb.

These images demonstrate that the three regimes produce quite different hole morphologies and depths. The single-pulse regime creates highly tapered holes with a wide entrance diameter and a rough inner surface. Moreover, the depth starts saturating around 300 ms corresponding to 300 pulses with each an energy of 140 µJ at a depth value of about 160 µm. While switching to MHz bursts (2 ppb), we observe a higher saturation depth of about 280 µm. The corresponding inner walls appear smoother than for repetitive single pulses. With longer MHz bursts (8 ppb), the inner walls become even smoother, probably owing to the lower intensity of the individual pulses and to the onset of a beneficial heat accumulation effect. Finally, the GHz-burst regime provides the best hole quality of the investigated regimes, as we can observe a more cylindrical hole geometry and a lower level of cracks. Moreover, in this regime, the holes become longer than in the MHz-burst mode for long drilling times as we will show below.

**Figure 3:**
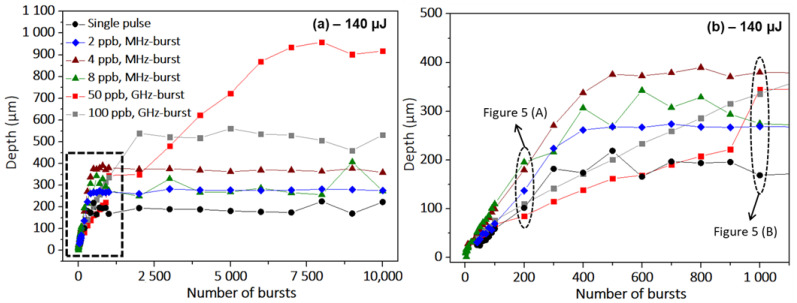
Evolution of the hole depth as a function of the number of bursts (pulses) applied to the sample in sodalime for the single-pulse regime, MHz-burst regime with 4 and 8 ppb at 40 MHz, and GHz-burst regime for 50 and 100 ppb at 1.28 GHz (**a**). Zoom on the evolution of the depth as a function of the number of bursts (pulses) for the beginning of the graph as indicated by the dashed rectangle (**b**). The burst (pulse) energy is fixed at 140 µJ and the burst (pulse) repetition rate is fixed at 1 kHz. The encircled data points correspond to the microscope images shown below.

The evolution of the hole depth as a function of the number of bursts (pulses in SPR), and thus drilling time, on the full range of this study (drilling times up to 10 s) is depicted in Figure 3. As all these experiments were also carried out at a pulse or burst repetition rate of 1 kHz, 1000 bursts correspond to a drilling time of 1 s. The black dots correspond to the single-pulse regime, the brown and green triangles correspond to the MHz-burst mode with 4 ppb and 8 ppb, respectively, and the blue lozenges correspond to the MHz-burst regime with 2 ppb. The red and grey squares correspond to the GHz-burst mode with 50 pulses per bursts and 100 pulses per bursts, respectively. No error bars are visible on these graphs as the uncertainty in the depth measurements is negligible (lower than ±5 µm).

**Figure 4:**
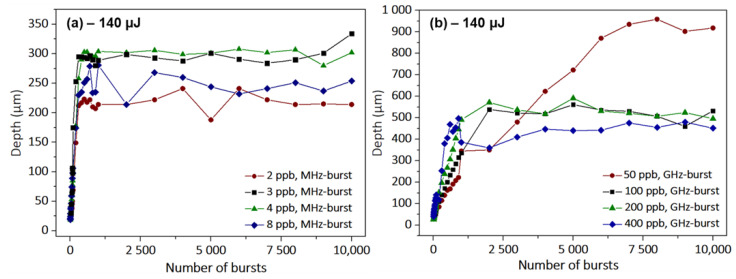
Evolution of the hole depth as a function of the number of bursts applied to the sodalime sample for the MHz-burst regime with 2, 3, 4, and 8 ppb at a 40 MHz repetition rate for a burst energy of 96 µJ (**a**), and for the GHz-burst regime with 50, 100, 200, and 400 ppb at a 1.28 GHz repetition rate for a burst energy of 144 µJ (**b**). The burst repetition rate is 1 kHz in both cases.

In these graphs, we observe a very different behavior of the three regimes regarding the evolution of the depth as a function of the number of pulses/bursts, respectively. Regarding the maximum achievable hole depth corresponding to the values where the graphs saturate, we observe that the GHz-burst leads by far to the highest depth values. For a GHz-burst with 50 ppb, the maximum depth is up to five times more important than the one obtained with repetitive single pulses. Note that longer drilling times are linked to higher hole depths. However, increasing the number of ppb does not necessarily lead to deeper holes. In GHz-burst mode with 100 ppb, the maximum depth drops by a factor of two with respect to the GHz-bursts with 50 ppb. Obviously, there exists an optimum value of the number of ppb to reach the highest hole depth. This behavior reflects that the burst length is subject to a compromise. Increasing the burst length, and thus the ppb, in GHz-burst mode allows for a longer time of beneficial heat accumulation during the burst. However, increasing the number of ppb at a constant burst energy implies a spreading over more and more pulses. Thus, a longer burst means less laser intensity per pulse within the burst and, consequently, a less effective non-linear absorption. This results in a lower effective burst energy reaching the tip of the hole which will eventually not be enough to generate an ablation process and the drilling process will stop, which corresponds to the saturation of the hole depth. For the MHz-burst regime, we can see the same general trend, the depth obtained at 4 ppb is more important than the ones for 2 ppb and 8 ppb, suggesting that there might be an optimum number of pulses per bursts or laser intensity. In Figure 3b, we display a zoom as indicated by the dashed rectangle of the first part of the graph which is also worth discussing. As was extensively reported in a recent article [33], the GHz-burst mode drilling is driven by a three-stage behavior (i) surface ablation, (ii) deep ablation, and (iii) saturation of the drilling, which can also be observed in these graphs. In addition, these graphs display an increase in the ablation rate from 50 ppb to 100 ppb as can be derived from the slopes of the graphs in Figure 3 and Figure 4. This observation is in agreement with the studies reported in the literature [22,23,24,25]. For the repetitive single-pulse regime and the MHz-burst regime, the behavior appears to be slightly different and seems to display only two stages: firstly, the linear increase in the depth as a function of the number of pulses/bursts, respectively, and secondly, the saturation of the hole depth, where the drilling process is over. One can reasonably assume that the saturation stage results from the same phenomena as in the GHz-burst mode, which means losses in the beam propagation towards the tip of the hole resulting in too little energy reaching the tip of the hole to generate ablation. However, in the beginning of the graph, for a low number of pulses/bursts, respectively, we can assume that the surface ablation process is skipped due to the high energy of the pulses. The encircled data points correspond to the microscope images that are shown below for a comparison of the hole morphologies.

In order to optimize the hole depth saturation value, we investigated the dependence on the number of pulses per burst in both MHz-burst (a) and GHz-burst regimes (b) for the optimized burst energy values. The resulting graphs are depicted in Figure 4.

Both of these graphs confirm the hypothesis that there is an optimum number of ppb that allows for maximizing the achievable hole depth. For MHz-bursts, it appears that for sodalime the optimum number of pulses per burst is around 4, while in the GHz-burst regime the best value leading to the deepest holes is clearly 50 ppb in the tested configurations in this experiment. This confirms the observation that in the GHz-burst mode, a compromise has to be found concerning the burst length. On the one hand, longer bursts enable more beneficial heat accumulation and thus deeper holes. On the other hand, increasing the burst length implies a spreading of the burst energy over more individual pulses and a decrease in non-linear absorption resulting in lower saturation values for the hole depth. Interestingly, in the MHz-burst mode, the number of ppb appears to have a limited effect on the ablation rate given by the slope of the graph (drilling process before saturation). Indeed, in this regime, each pulse within the burst disposes of an energy exceeding the ablation threshold of sodalime [36]. Thus, increasing the number of pulses per burst at a constant total energy will not increase the ablation rate as the pulse-to-pulse delay within the MHz-burst is much higher than the heat diffusion time of the material [26]. On the other hand, the GHz-burst regime displays a very different behavior regarding the evolution of the ablation rate as a function of the number of pulses per bursts. As shown in Figure 4b, the ablation rate per burst increases with the number of pulses per burst. The slopes of the two first stages increase from 0.7 µm/burst for the surface ablation rate at 50 ppb up to 2.5 µm/burst at 400 ppb. The different ablation rates, as well as the average depths of saturation, for all of the parameters investigated during this study are summarized in Table 1. In this table, Rate 1 corresponds to the surface ablation rate and Rate 2 to the deep ablation rate with respect to the GHz-burst regime drilling process.

This table shows that the single-pulse regime displays a rather low ablation rate, nearly two times lower than the ablation rate of the MHz-burst regime. Moreover, the maximum average depth of saturation for comparable energy is much lower than the one achievable in the MHz-burst regime (by a factor of 2 for 5 ppb) and in the GHz-burst regime (by a factor of 4.5 for 50 ppb). As can be deduced from the graphs, the ablation rate in the MHz-burst regime is rather constant over the increasing number of pulses and much higher than in the single-pulse regime. Regarding the depth, there is indeed an optimum number of pulses per burst that allows for deeper drillings, which corresponds in this case to 5 ppb. For the GHz-burst regime, we show the two ablation rates that can be estimated from the graphs by linear fits. The surface ablation rate is much higher than the deep ablation rate, probably due to a screening effect from the ablation plume [33]. One can notice that the deep ablation rate is much lower for 50 and 100 ppb than for longer bursts. This can be explained by a smaller ablation plume inducing a reduced screening effect within the burst, as the energy is repartitioned between a higher number of pulses. Therefore, a much longer burst gives higher deep ablation rates. However, as mentioned before, the hole saturation depth is subject to a compromise in the number of pulses per burst depending on the material and the laser energy. Note that in the single-pulse and in the MHz-burst regimes, higher ablation rates lead to deeper holes. In contrast, in the GHz-burst regime, smaller ablation rates are linked to higher values of hole depth meaning that slower drilling allows for achieving deeper holes.

**Figure 5:**
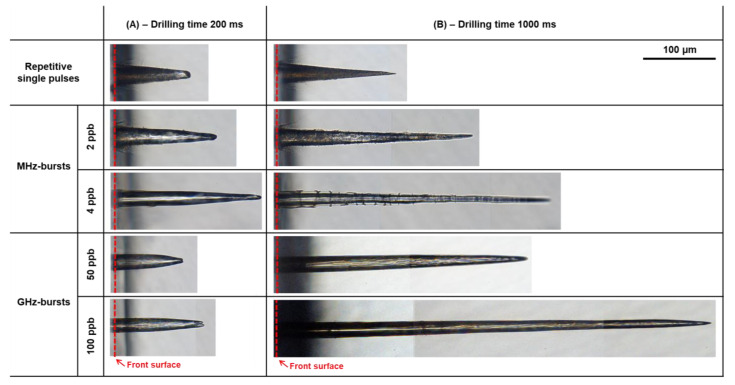
Sideview microscope images of the holes drilled in sodalime taken with a 50X microscope objective with repetitive single pulses, in MHz-burst mode with 2 ppb and 4 ppb and in GHz-burst mode with 50 ppb and 100 ppb I. The left column (**A**) depicts holes for a drilling time of 200 ms corresponding to 200 bursts (pulses) and the right column (**B**) depicts holes for a drilling time of 1 s corresponding to 1000 bursts (pulses in SPR). The burst (pulse in SPR) energy and the repetition rate are fixed at 140 µJ and 1 kHz, respectively.

In order to evaluate the hole and inner walls morphology, we took high-resolution images of the holes with a microscope objective 50X (see Figure 5). The images displayed in this figure correspond to holes drilled with 200 bursts (pulses in SPR) for the left column (A) and with 1000 bursts (pulses in SPR) for the right column (B) of Figure 5. In this figure, we observe that the single-pulse regime and the MHz-burst regime with 2 ppb produce similar inner walls and hole morphologies. Indeed, in Figure 5, the inner walls of the holes appear rough and the holes are tapered. In addition, these two laser configurations are the only ones to display a collar at the surface, attesting to a pulse energy on the material that is too high and may lead to modifications in its properties and eventually to a lowered surface quality.

For MHz-bursts with 4 ppb, the hole quality appears to change. The inner walls are clearly smoother but cracky. It is possible that for this regime each individual pulse within the burst is still too energetic. Note that the hole morphology in the MHz-burst regime with 8 ppb is very similar and not shown here. Further, one may notice that the holes are still conical but tend towards the GHz-burst regime. For all drilling times, the GHz-burst mode produces more cylindrical holes than the two previous regimes and a better surface quality.

These observations support the idea that the MHz-burst interaction is driven by laser intensity, where the optimum intensity is a compromise between non-linear absorption and cumulative effects, whereas the GHz-burst regime is mainly driven by heat accumulation. On the contrary, in the MHz-burst regime, the cooperative effect between pulses within the burst has a lower impact since the pulse-to-pulse delay is much longer than the mean heat-relaxation time of the material [26]. Moreover, despite the fact that for a 200 ms drilling time the holes in this regime are less deep than the ones in the MHz-burst mode, at a 1 s drilling time the tendency is inverted. The hole obtained with 100 ppb has a depth of 600 µm compared to the 385 µm of the MHz-burst with 4 ppb. In addition, one should notice the excellent quality of the holes drilled in GHz-burst mode and that the cylindrical shape is kept even for deep drillings.

### 3.2. GHz-Burst Drilling of Fused Silica: Towards Extreme Aspect Ratios

**Figure 6:**
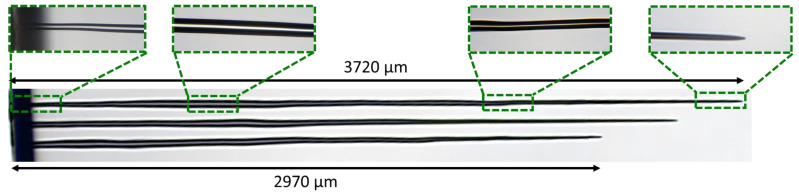
Holes drilled in fused silica in GHz-burst mode with bursts of 250 µJ and a number of 70 ppb at repetition rates of 100 kHz, 50 kHz, and 20 kHz (from the top to the bottom), respectively, and a drilling time of 5 s. The green rectangles are zooms taken with a 50X objective on the microscope.

In this section, we report on tuning the operating parameters in order to obtain extreme hole geometries, in terms of depth and aspect ratio. Since the burst repetition rate has been identified as a critical parameter to obtain high aspect ratios, we have chosen to work on fused silica, a material that can stand higher values of repetition rate (up to 100 kHz) without detrimental side-effects or hole collapse [34]. Moreover, fused silica is a standard material in many applications. Furthermore, we have also considered the numerical aperture, since this parameter has a direct influence on the beam propagation towards the tip of the hole by multiple reflections on the inner walls of the hole [33]. Therefore, the numerical aperture was reduced from 0.14 to 0.09 in order to benefit from maximum reflections under grazing incidence. Note that the beam focus stays at the sample surface and neither the sample nor the beam focus are moved. Contrary to the previous section, we used classical gain-depleted GHz-bursts which recently gave excellent drilling results on sodalime [35]. GHz-burst drilling was performed with bursts of 250 µJ burst energy and 70 ppb at 20 kHz, 50 kHz, and 100 kHz, respectively. The resulting holes after a drilling time of 5 s are depicted in Figure 6. Note that we voluntarily chose a relatively long drilling time with respect to the repetition rates to ensure that the drilling process stops from internal causes (saturation) and not from a too short drilling time.

In this figure, we observe extremely deep holes with an excellent quality. The diameters at the entry of the holes were measured just after the shadows arising from the surface. Note that the hole is slightly smaller at this point, but it was not refilled after drilling. From the top to the bottom, the diameters are 25 µm, 30 µm, and 34 µm, respectively, and the corresponding depths are 3.72 mm, 3.40 mm, and 2.97 mm, respectively. These values correspond to an aspect ratio as high as 150 for the deepest hole, which is the highest aspect ratio reported thus far with femtosecond GHz-burst drilling. The zooms taken at different depths of the hole attest that the inner walls retain an excellent quality, they are glossy and crack-free all along the hole. Moreover, one can notice that there is no heat-affected zone whatsoever, despite the relatively high burst repetition rate and burst energy.

## 4. Conclusions

In this study, we investigated and compared the microdrilling process in sodalime with repetitive single pulses, as well as in MHz-burst and GHz-burst regimes. We varied the number of pulses within the burst and the drilling time. The results are discussed in terms of ablation rate, hole depth, and inner walls morphology.

We observe that the ablation rate increases with the number of pulses in the GHz-burst regime, whereas it is almost constant in the MHz-burst regime. Furthermore, the maximum achievable depth increases with decreasing burst length in the GHz-burst regime until reaching an optimum value which is subject to a compromise. Interestingly, in this regime, the lower the ablation rate the longer the holes. The repetitive single-pulse and the short MHz-burst (2 ppb) regimes lead to short and highly tapered holes, with rough inner walls. Longer MHz-bursts (8 ppb) and the GHz-burst regime give elongated holes with smoother inner walls. The best drilling quality is clearly obtained in the GHz-burst regime where the holes are almost cylindrical and show crack-free and glossy inner walls.

Finally, we investigated the drilling process capability in terms of extreme depth and aspect ratio by tuning the burst repetition rate and the numerical aperture. In fused silica, the deepest crack-free hole that could be drilled was 3.7 mm long with a 25 µm entrance diameter corresponding to an aspect ratio of 150, which is the highest aspect ratio reported thus far with femtosecond GHz-burst drilling.

## Figures and Tables

**Table 1:** Drilling rates and average saturation depths in sodalime for a burst (pulse for SPR) energy of 140 µJ for the single-pulse regime, the MHz-burst regime for 2 to 8 ppb, and in the GHz-burst regime for 50 to 400 ppb.

	SPR	MHz-Burst Mode							GHz-Burst Mode (Flat Bursts)			
Nbr of Pulses	1	2	3	4	5	6	7	8	50	100	200	400
Rate 1 (µm/burst)	/	/	/	/	/	/	/	/	0.7	1.1	2	2.5
Rate 2 (µm/burst)	0.55	0.75	0.75	0.8	1	0.95	1	1	0.125	0.22	0.48	0.8
Avg. depth (µm)	190	280	334	390	400	350	320	300	900	550	490	450

## Data Availability

Data underlying the results presented in this paper are not publicly available at this time but may be obtained from the authors upon reasonable request.
